# Nisin probiotic prevents inflammatory bone loss while promoting reparative proliferation and a healthy microbiome

**DOI:** 10.1038/s41522-022-00307-x

**Published:** 2022-06-07

**Authors:** Li Gao, Ryutaro Kuraji, Martin Jinye Zhang, April Martinez, Allan Radaic, Pachiyappan Kamarajan, Charles Le, Ling Zhan, Changchang Ye, Hélène Rangé, M. Reza Sailani, Yvonne L. Kapila

**Affiliations:** 1 Orofacial Sciences Department, School of Dentistry, University of California, San Francisco (UCSF), San Francisco, CA USA; 2 Department of Periodontology, Hospital of Stomatology, Guanghua School of Stomatology, Sun Yat-sen University, Guangdong Provincial Key Laboratory of Stomatology, Guangzhou, China; 3 Department of Life Science Dentistry, The Nippon Dental University, Tokyo, Japan; 4 Department of Periodontology, The Nippon Dental University School of Life Dentistry at Tokyo, Tokyo, Japan; 5 Oralome, Inc, 1700 4th Street, Byers Hall Suite 214, San Francisco, CA USA; 6 State Key Laboratory of Oral Diseases, National Clinical Research Center for Oral Diseases, Department of Periodontology, West China School of Stomatology, Sichuan University, Chengdu, China; 7 Université Paris Cité, Faculty of Health, Department of Periodontology, URP2496 Orofacial Pathologies, Imaging and Biotherapies Laboratory, Montrouge and Paris Center for Microbiome Medicine, PaCeMM, FHU, Hôpital Rothschild, APHP, Paris, France

**Keywords:** Antimicrobials, Clinical microbiology

## Abstract

Dysbiosis of the oral microbiome mediates chronic periodontal disease. Realignment of microbial dysbiosis towards health may prevent disease. Treatment with antibiotics and probiotics can modulate the microbial, immunological, and clinical landscape of periodontal disease with some success. Antibacterial peptides or bacteriocins, such as nisin, and a nisin-producing probiotic, *Lactococcus lactis*, have not been examined in this context, yet warrant examination because of their biomedical benefits in eradicating biofilms and pathogenic bacteria, modulating immune mechanisms, and their safety profile in humans. This study’s goal was to examine the potential for nisin and a nisin-producing probiotic to abrogate periodontal bone loss, the host inflammatory response, and changes in oral microbiome composition in a polymicrobial mouse model of periodontal disease. Nisin and a nisin-producing *Lactococcus lactis* probiotic significantly decreased the levels of several periodontal pathogens, alveolar bone loss, and the oral and systemic inflammatory host response. Surprisingly, nisin and/or the nisin-producing *L. lactis* probiotic enhanced the population of fibroblasts and osteoblasts despite the polymicrobial infection. Nisin mediated human periodontal ligament cell proliferation dose-dependently by increasing the proliferation marker, Ki-67. Nisin and probiotic treatment significantly shifted the oral microbiome towards the healthy control state; health was associated with *Proteobacteria*, whereas 3 retroviruses were associated with disease. Disease-associated microbial species were correlated with IL-6 levels. Nisin or nisin-producing probiotic’s ability to shift the oral microbiome towards health, mitigate periodontal destruction and the host immune response, and promote a novel proliferative phenotype in reparative connective tissue cells, addresses key aspects of the pathogenesis of periodontal disease and reveals a new biomedical application for nisin in treatment of periodontitis and reparative medicine.

## Introduction

Periodontitis, a chronic inflammatory disease of the hard and soft tissues that support teeth, is characterized by clinical attachment loss and alveolar bone loss. Periodontal disease is prevalent both in developed and developing countries and affects about 40–50% of the global population, making it a high public health concern^1,2^. It is associated with several systemic diseases, such as diabetes mellitus, cardiovascular diseases, adverse pregnancy outcomes, rheumatoid arthritis, respiratory infections, Alzheimer’s disease, and cancer^3–6^. A dysbiotic oral microbiota is the initiating factor in the etiology of periodontitis, which leads to a dysregulated host immune response^7,8^. These dysbiotic oral microbes live in oral biofilms.

Oral biofilms are microbial communities with increased resistance to antimicrobial agents and elevated levels of virulence factors compared with planktonic bacteria. Among the biofilm-associated microbiota, the bacterial species *Porphyromonas gingivalis, Treponema denticola, Tannerella forsythia*, and *Fusobacterium nucleatum* have been strongly implicated in the development of periodontal disease^9,10^. Although novel sequencing methods are beginning to reveal other important microbes associated with periodontal disease and oral biofilm formation^11,12^, these four pathogens not only contribute to periodontal disease via a variety of mechanisms and virulence factors, but they also invade other organs and tissues^13^, induce systemic infection, and play roles in the pathogenesis of cancers, cardiovascular diseases, metabolic diseases, and Alzheimer’s disease^6,14–17^. Therefore, controlling periodontal pathogens and maintaining a healthy oral biofilm is important in addressing the global burden of periodontal disease.

Scaling and root planing (SRP) is the gold standard for treatment of periodontitis, which is effective in removing plaque and calculus accretions on the tooth surface. Although this therapy adequately lowers bacterial counts, recolonization by periodontopathogens is a major problem in maintaining long-term efficacy for periodontitis^18^. Considering the limitations of SRP, local and systemic antibiotic administration can help address these shortcomings^19,20^. However, antibiotic therapy may trigger gastrointestinal side effects^21^, bacterial resistance and allergic reactions^22–24^. For this reason, the administration of beneficial bacteria in the form of probiotics can be a valuable adjunct to SRP in the treatment of periodontitis. According to the World Health Organization (2012), probiotics are defined as ‘live microorganisms that, when administered in adequate amounts, confer a health benefit on the host’^25^. Recent publications have demonstrated the potential benefit of probiotic administration for reducing periodontopathogenic bacteria, regulating immune response, and improving the clinical signs of the disease, suggesting a promising role for probiotics in enhancing periodontal health^26–37^.

The probiotic *Lactococcus lactis* produces one of the most widely used bacteriocins, known as nisin. Nisin, a lantibiotic, is the first and only bacteriocin approved for use in food preservation by the US Food and Drug Administration. Lantibiotics, a subgroup of bacteriocins produced by gram positive bacteria, are characterized by the presence of the unusual thioether amino acids lanthionine and 3-methyllanthionine generated through posttranslational modification^38^. Nisin is used globally and approved by the WHO for the same application. Nisin also has potential as a therapeutic agent in medical, dental, and veterinary applications^39,40^. Howell et al.^41^ found that nisin was effective in the reduction of plaque build-up and gingivitis in a beagle dog model. Cunha et al.^42^ also reported the potential role of nisin in the control of periodontal disease in dogs. Our published research^43,44^ demonstrated that nisin effectively abrogates the growth of planktonic pathogenic bacteria and biofilm-encased bacteria associated with caries, periodontal disease, and persistent endodontic infections without inducing cytotoxicity to human oral cells. Nisin also resets pathogenic oral biofilms towards control/healthy levels in vitro^45^. Although oral delivery of nisin can alter the gut microbiota in mice^46^, and change the oral microbiomes in healthy rats and dogs^47,48^, there is no research on the effect of nisin-producing *L. lactis* on the oral microbiome (bacteriome and virome) in the context of periodontal disease. Taken in aggregate, these data provide the basis for the current investigation. In this study, a polymicrobial mouse model of periodontal disease^12^ was induced by oral infection with *P. gingivalis*, *T. denticola*, *T. forsythia*, and *F. nucleatum*, and employed to examine the effects of nisin and the nisin-producing probiotic *L. lactis* in abrogating periodontal bone loss and modulating the composition of the oral microbiome and inflammatory landscape.

## Results

### Polymicrobial oral infection is reduced by nisin or nisin-producing probiotic

**Fig. 1:**
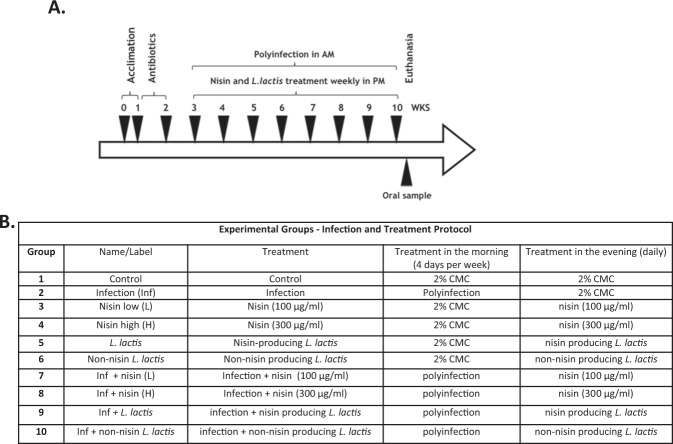
Mouse treatment procedure and oral sample collection timeline. The schematic diagram of the experimental design is shown in **A** and the treatment protocol of each group is shown in **B**. Polymicrobial infections were carried out in the morning for 4 consecutive days once per week from the 3rd to the 10th week. Nisin and *L. lactis* were administered every day in the evening from the 3rd to the 10th week. Oral swab samples were collected at 8 weeks following the initial infection. Blood and tissue specimen collection was performed at euthanasia following 8 weeks of infection.

**Fig. 2:**
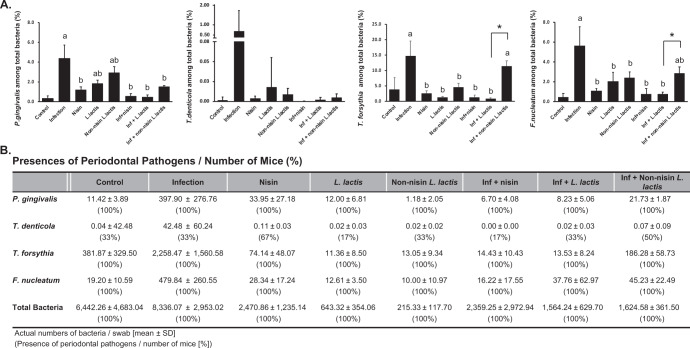
Polymicrobial oral infection is reduced by nisin or nisin-producing probiotic treatment. Oral swab samples were collected at 8 weeks after polymicrobial infection. DNA was isolated and purified from the swab samples of eight groups (Control, Infection, Nisin (H), *L. lactis*, Non-nisin *L. lactis*, Infection + nisin (H), Infection + *L. lactis* and Infection + Non-nisin *L. lactis;* *n* = 6 mice per group). The total bacteria were quantified by standard real-time PCR using primers corresponding to 16S ribosomal RNA. **A** The data are shown as a percentage of each pathogen (*P. gingivalis*, *T. denticola*, *T. forsythia, or F. nucleatum*) among total bacteria. Data represent the means ± standard deviation from six mice per group.Statistical significance was determined using an ANOVA followed by a Tukey’s test. The difference in variance with a *p*-value of <0.05 was considered significant. (a) The difference in percentage of the pathogen was significant (*p* < 0.001) compared to the Control group. (b) The difference in percentage of the pathogen was significant (*p* < 0.01) compared to the Infection group. *, the difference in percentage of the pathogen between the two groups was significant (*p* < 0.05). **B** The table demonstrates the number of detected bacteria and detection frequency (%) of periodontal pathogens in each swab from each mouse relative to the number of collected samples.

A PCR-based approach was used to evaluate the ability of nisin and a nisin-producing probiotic *L. lactis* to modulate the oral infection consisting of periodontal pathogens in a polymicrobial infection mouse model (Fig. 1A, B). Oral swab results indicated that all four bacteria were detectable at 8 weeks post infection (Fig. 2A). In the infection group, *P. gingivalis*, *T. forsythia*, and *F. nucleatum* were present at significantly higher levels than the control group (*p* < 0.001). Similarly, *T. denticola* showed a trend toward higher levels in the infection group, but this was not significantly different from the control group. Treatment with nisin or the nisin-producing *L. lactis* probiotic markedly decreased the number of *P. gingivalis*, *T. forsythia*, and *F. nucleatum* compared to the infection group (*p* < 0.01). In contrast, the non-nisin-producing probiotic group showed that *T. forsythia* and *F. nucleatum* didn’t recover back to control levels; and these levels were significantly higher compared to the nisin-producing probiotic group (*p* < 0.05).

In addition, the frequency of mice exhibiting infection differed depending on the pathogens (Fig. 2B). *P. gingivalis* as well as *T. forsythia* and *F. nucleatum* were present in all mice in all groups, whereas *T. denticola* was present in much fewer mice across all groups.

### Alveolar bone loss parameters were significantly inhibited in mice treated with nisin or nisin-producing probiotic

**Fig. 3:**
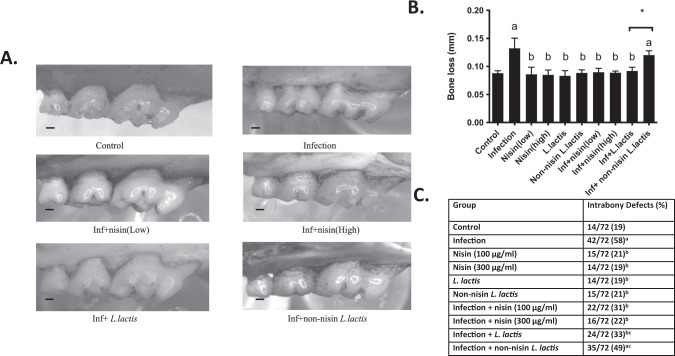
Alveolar bone loss is significantly abrogated with nisin or nisin-producing probiotic treatment. **A** Representative images of alveolar bone loss on the palatal surfaces of maxillary molars in six groups (Control, Infection, Infection + nisin (L), Infection + nisin (H), Infection + *L. lactis* and Infection + Non-nisin *L. lactis*). Scale bar represents 0.2 mm. **B** The graph represents alveolar bone loss in all ten groups. Data represent the means ± standard deviation from six mice per group. For each mouse, alveolar bone loss was calculated as the average from 28 sites (3 sites on the first molar, 2 sites on the second molar, and 2 sites on the third molar, on both sides of the left maxilla and mandible). Statistical significance was determined using Student’s *t*-test between two independent groups. The difference in variance with a *p*-value of <0.05 was considered significant. (a) The difference in alveolar bone loss was significant (*p* < 0.05) compared to the Control group. (b) The difference in alveolar bone loss was significant (*p* < 0.05) compared to the Infection group. *, the difference in alveolar bone loss between the two groups was significant (*p* < 0.05). **C** The percentage of intrabony defects was calculated as the number of tooth surfaces containing periodontal intrabony defects out of total tooth surfaces. For each group, there were a total of 72 tooth surfaces (6 mice, 36 molars, 72 sides (buccal, palatal/lingual)). A chi-square test was used for analysis of the percentage of intrabony defects, the difference in variance with a *p*-value of <0.05 was considered significant. (a) The difference in the percentage of intrabony defect was significant (*p* < 0.05) compared to the Control group. (b) The difference in the percentage of intrabony defect was significant (*p* < 0.05) compared to the Infection group. (c) There was no significant difference in the percentage of intrabony defect between the Infection + *L. lactis* group and Infection + non-nisin *L. lactis* group (*p* > 0.05).

A polymicrobial infection mouse model of periodontal disease was used to evaluate the ability of nisin and a nisin-producing probiotic *L. lactis* to modulate periodontal bone loss. After 8 weeks of inoculation/infection with periodontal pathogens (*P. gingivalis*, *T. denticola*, *T. forsythia* and *F. nucleatum*), mice treated simultaneously with nisin or the nisin-producing probiotic *L. lactis* exhibited significantly less bone loss compared to the infection group (Fig. 3A, B). Treatment with either high or low concentrations of nisin both showed significant rescue effects and significantly diminished bone loss in the presence of infection. The non-nisin producing *L. lactis* probiotic was unable to prevent the bone loss in the infected group.

The presence of alveolar intrabony defects were also evaluated following treatment. Nineteen percent of control uninfected sites showed a baseline level of intrabony defects compared to 58% of infected sites (Fig. 3C). Nisin (low or high concentrations) and the nisin-producing probiotic significantly decreased the number of sites that exhibited intrabony defects; 31%, 22%, and 33%, respectively. The non-nisin producing probiotic was unable to significantly prevent the development of intrabony defects; however, the percentage of sites (49%) that exhibited defects was lower than that of infected sites (58%). Although, the comparison between the Infection + *L. lactis* group (33%) and Infection plus non-nisin *L. lactis* group (49%) showed no significant difference (*p* > 0.05), the Infection plus non-nisin *L. lactis* group exhibited higher numbers. However, the Infection plus *L. lactis* group was significantly different from the Infection group, but the Infection plus non-nisin *L. lactis* group was not.

### Systemic host antibody response against periodontal pathogens is attenuated with nisin or nisin-producing probiotic

**Fig. 4:**
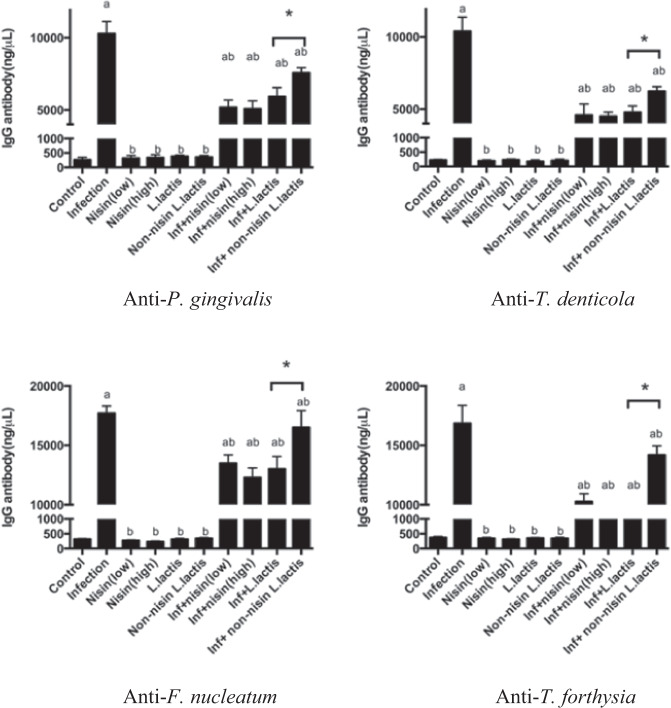
Host antibody response against periodontal pathogens is significantly abrogated with nisin or nisin-producing probiotic treatment. Serum IgG antibody levels to *P. gingivalis*, *T. denticola*, *T. forsythia*, and *F. nucleatum* in all ten groups is shown. Data represent the means ± standard deviation from six mice per group. Statistical significance was determined using Student’s *t*-test between the two independent groups. The difference in variance with a *p*-value of <0.05 was considered significant. (a) The difference in serum IgG antibody levels was significant (*p* < 0.05) compared to the Control group. (b) The difference in serum IgG antibody levels was significant (*p* < 0.05) compared to the Infection group. *, the difference in serum IgG antibody levels between the two groups was significant (*p* < 0.05).

To evaluate the host response to the polymicrobial infection, serum antibody levels to the 4 periodontal pathogens were evaluated using an ELISA. Control infected mice showed a significant antibody response to all 4 periodontal pathogens compared to the uninfected control mice (Fig. 4). Nisin (low or high concentration) and the nisin-producing probiotic significantly decreased the antibody response in the infected mice. The non-nisin producing *L. lactis* was also able to decrease the antibody response to the periodontal pathogens, however, the effect was not as significant as that observed with the nisin-producing probiotic.

### Nisin or nisin-producing probiotic prevent an influx of inflammatory cells into the periodontal complex upon polymicrobial infection

**Fig. 5:**
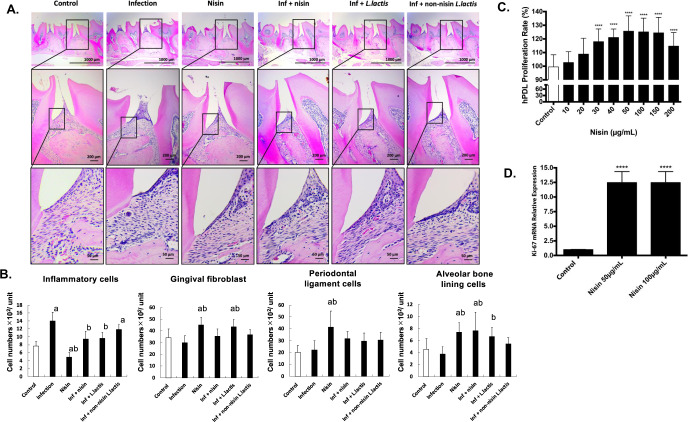
Nisin or nisin-producing probiotic prevent an influx of inflammatory cells into the periodontal complex, and promote increases in host reparative periodontal cells. Histological examination of periodontal inflammation in the interproximal area between the first and second maxillary molars was performed in six groups (Control, Infection, nisin, Infection + nisin (H), Infection + *L. lactis* and Infection + Non-nisin *L. lactis*). **A** Representative histological images of morphologic changes within the periodontal tissues using HE staining of sagittal sections. **B** The bar graphs demonstrate the number of inflammatory cells and host periodontal cells per 1.0 mm^2^ of connective tissue in the maxillary specimens. In three tissue sections per mouse specimen, the number of inflammatory cells, gingival fibroblasts in connective tissues adjacent to the gingival epithelium, number of periodontal ligament cells, and alveolar bone lining cells were counted within a square field (100 × 100 μm) between first and second molars. Data represent the means ± standard deviation from three mice per group. Statistical significance was determined using an ANOVA followed by a Tukey’s test. The difference in variance with a *p*-value of <0.05 was considered significant. (a) Significantly different compared to the control group (*p* < 0.05); (b) significantly different compared to the infection group (*p* < 0.05). **C** Nisin treatment promoted human periodontal ligament cell proliferation (****p < 0.0001). **D** Nisin treatment promoted Ki-67 gene expression in human periodontal ligament cells (****p < 0.0001). Data represent the means ± standard deviation of three independent experiments. Statistical significance was determined using Student’s *t*-test between two independent groups. The difference in variance with a *p*-value of <0.05 was considered significant (**C** and **D**).

To evaluate nisin’s ability to alter the local host inflammatory response in the context of periodontal disease, we evaluated the inflammatory cell infiltrate and morphologic changes within the periodontal tissues using hematoxylin and eosin staining of sagittal sections (Fig. 5A). In the control group, few inflammatory cells were observed in the gingival connective tissue just below the thin junctional epithelium. In contrast, the gingival tissues from the polymicrobial infection group exhibited an infiltration of numerous inflammatory cells (*p* < 0.001; Fig. 5B) and deep pseudo periodontal pocket formation with epithelial hyperplasia and rete ridge elongation; note the height of the gingival margin in the infection group relative to the other treatment groups. Treatment with nisin and the nisin-producing probiotic *L. lactis* significantly decreased the inflammatory cell infiltrate in the infection group (*p* < 0.001). However, treatment with the non-nisin-producing *L. lactis* did not significantly decrease the inflammation compared to the infection group.

### Nisin or nisin-producing probiotic activate a proliferative phenotype in reparative connective tissue cells of the periodontium

Surprisingly, nisin and/or the nisin-producing *L. lactis* probiotic also markedly increased the number of fibroblast-like and osteoblast cells (gingival fibroblasts, periodontal ligament cells, alveolar bone lining cells) compared to the control and/or infection groups (*p* < 0.05; Fig. 5B). In contrast, application of the non-nisin-producing *L. lactis* did not significantly increase the number of gingival fibroblasts or alveolar bone lining cells. In vitro results showed that nisin (from 300 to 200 μg/ml) dose-dependently and significantly promoted human periodontal ligament cell proliferation, which is consistent with the findings in the mouse model (Fig. 5C). Furthermore, quantitative reverse transcription PCR (qRT-PCR) results showed significantly increased expression of the proliferation gene, Ki-67, in human periodontal ligament cells after nisin (50, 100 μg/ml) treatment (Fig. 5D). This is the first time that nisin or a probiotic have been shown to promote cell proliferation of reparative connective tissue cells in the context of a chronic inflammatory state.

### Nisin or nisin-producing probiotic abrogates the host inflammatory cytokine response to the periodontal pathogens

**Fig. 6:**
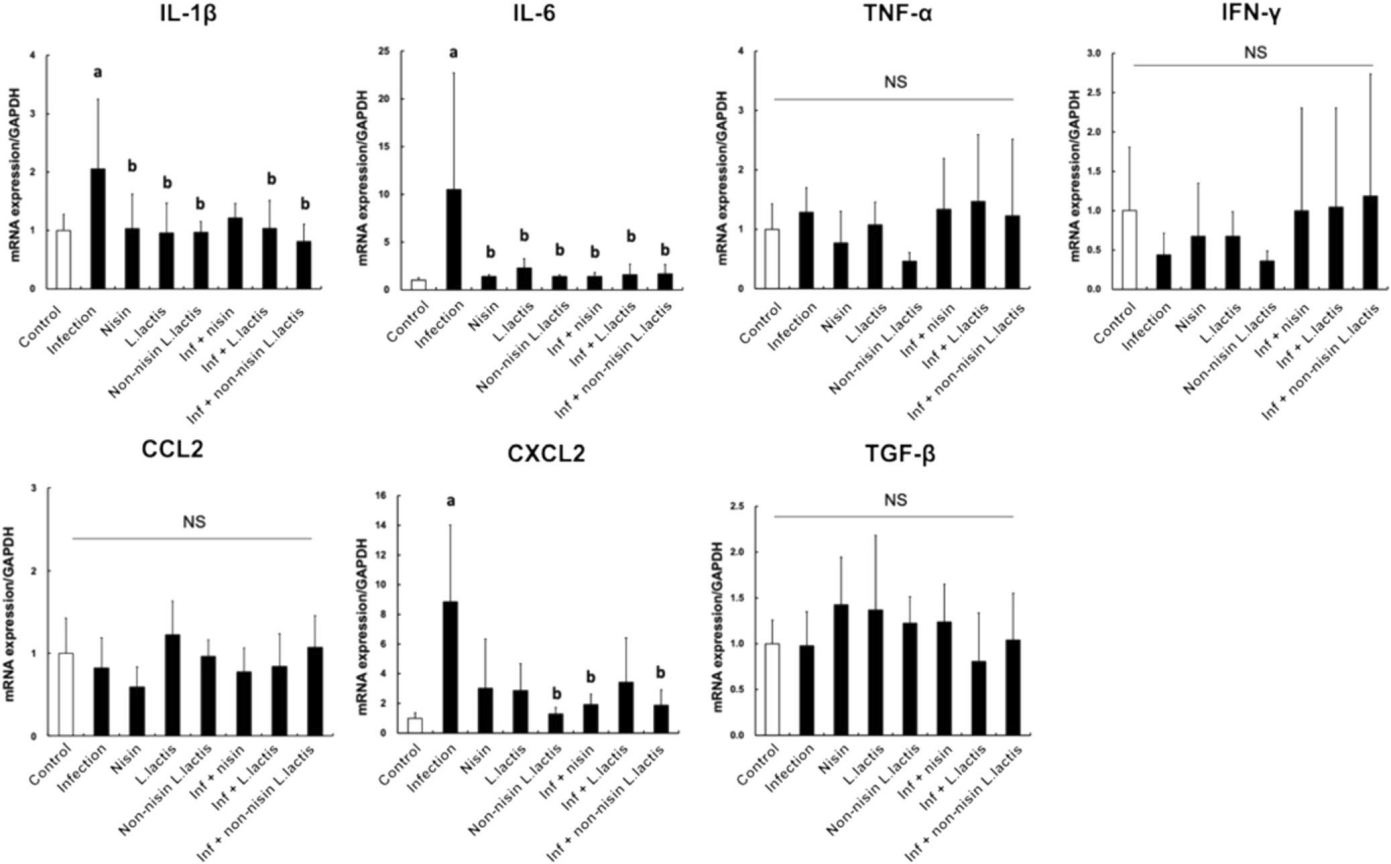
Nisin or nisin-producing probiotic abrogate the host inflammatory cytokine response in gingival tissues. To evaluate the immune cytokine profiles in gingival tissues, mRNA expression of IL-1β, IL-6, TNF-α, IFN-γ, CCL2, CXCL2, and TGF-β1 were measured by real-time PCR. The amount of mRNA in each reaction was normalized to GAPDH, which is a housekeeping gene. Data are shown as means ± standard deviation from six mice per group. Statistical significance was determined using Student’s *t*-test between two independent groups. The difference in variance with a *p*-value of <0.05 was considered significant. (a) *p* < 0.05 compared with the Control group. (b) *p* < 0.05 compared with the Infection group.

To examine the effect of nisin or nisin-producing probiotic on the host inflammatory response in the context of periodontal disease, the relative gene expression of inflammatory cytokines was assessed in gingival tissues by real-time PCR (Fig. 6). The infection group showed a significant upregulation in IL-1β, IL-6, and CXCL2; the latter is homologous to IL-8 in mice (*p* < 0.05). Treatment with nisin significantly reduced the expression of IL-6 and CXCL2, whereas the nisin-producing *L. lactis* significantly reduced the expression of IL-1β and IL-6 in the infected mice. The non-nisin producing *L. lactis* also suppressed the inflammatory response similar to the nisin- producing *L. lactis*, indicating that the *L. lactis* itself mediates an anti-inflammatory response. Other cytokines, namely TNF-α, IFN-γ, CCL2, and TGF-β, showed no significant changes following the polymicrobial infection or nisin treatment, although the anti-inflammatory cytokine TGF-β showed a trend toward higher levels with nisin treatment.

### Nisin and the nisin-producing probiotic promote a shift from a disease-associated microbiome toward a “healthy control” oral bacteriome and virome

**Fig. 7:**
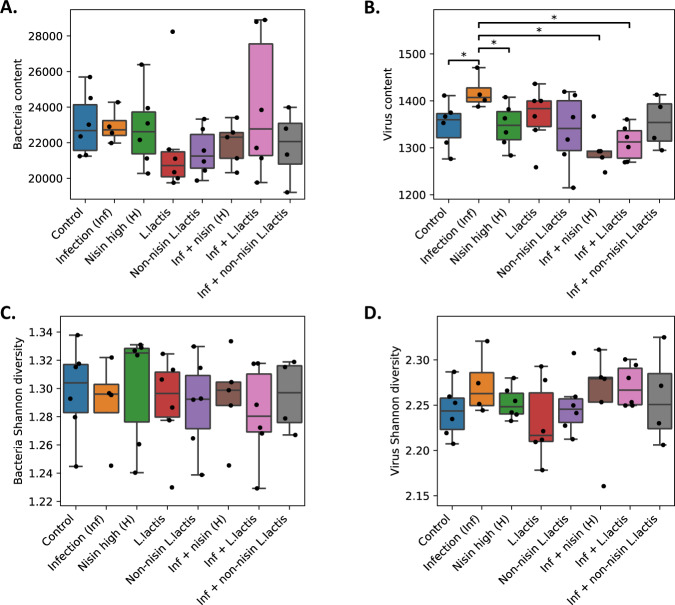
Comparison of bacterial and viral content and diversity scores across groups show differences in viral content upon infection that shift back with nisin treatment. The groups included Control, Infection, Nisin (H), *L. lactis*, Non-nisin *L. lactis*, Infection + Nisin (H), Infection + *L. lactis*, and Infection + Non-nisin *L. lactis*). **A**, **B** Bacterial and viral content in TPM and 95% confidence interval (CI) is shown across groups. In terms of bacterial content, there is no significant difference between groups. Statistical significance was determined using a two-sample *t*-test assuming equal variance of samples from the two groups. The difference in variance with a *p*-value of <0.05 was considered significant. *p < 0.05. In terms of viral content, the Infection group has significantly higher virus content than the Control group, Nisin group, Infection + Nisin (H) group, and Infection + *L. lactis* group. **C**, **D** Bacterial and viral Shannon diversity is shown for different groups. In terms of bacterial diversity, there is no significant difference between the groups. In terms of viral diversity, the Infection group has slightly higher but non-significant diversity than the Control and *L. lactis* groups. Data are presented as the box plots of six mice per group; the box shows the quartiles while the whiskers extend to show the rest of the distribution.

In order to assess how nisin and the nisin-producing probiotic modify the oral bacteriome and virome, and how it compares across infection and healthy groups, we conducted metagenome shotgun sequencing analysis of these different conditions. We compared the bacterial (Fig. 7A) and viral content (Fig. 7B) of groups treated with nisin, nisin-producing *L. lactis* probiotic, and non-nisin producing *L. lactis* with and without infection and compared against the control group and against the infection group. We observed significant differences in viral content across groups. However, we observed only minor differences in bacterial content. With regards to viral content for different groups, the infection group had significantly higher viral content than the control group (nominal *p*-value 0.041), nisin group (nominal *p*-value 0.032), infection plus nisin group (nominal *p*-value 0.0029), and infection plus *L. lactis* group (nominal *p*-value 0.0020). In concordance with the bacterial content, bacterial Shannon diversity for different groups showed no significant differences across groups (Fig. 7C). However, the viral diversity score was different across some groups. Specifically, the infection group was higher but not significantly different in diversity than the control group (nominal *p*-value 0.18) and *L. lactis* group (nominal *p*-value 0.16) (Fig. 7D).

**Fig. 8:**
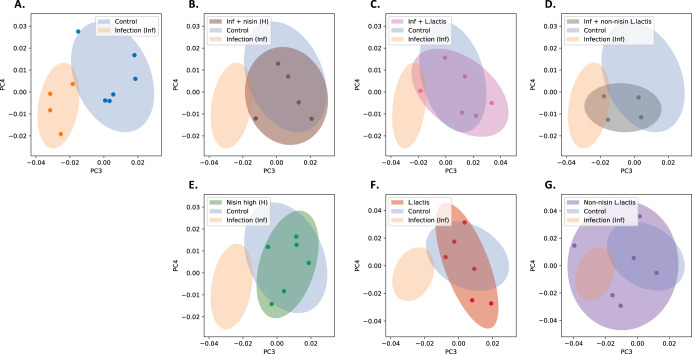
Principal coordinates analysis (PCoA) plots for microbiome composition of different groups showing nisin/ nisin-producing probiotic shift oral microbiome back toward healthy control levels following infection. (Control, Infection, Nisin (H), *L. lactis*, Non-nisin *L. lactis*, Infection + Nisin (H), Infection + *L. lactis*, Infection + Non-nisin *L. lactis*). **A** PC3 and PC4 separate the Control group from the Infection group. **B**–**G** Overlay each of the other groups on top of panel A, respectively. Among infected groups, those treated with nisin (panel B) and *L. lactis* (panel C) are similar to the Control group, while those treated with non-nisin *L. lactis* (panel D) is in the middle of the Control group and the Infection group. Among non-infected groups, those treated with nisin (panel E) and *L. lactis* (panel F) are similar to the Control group, while those treated with non-nisin *L. lactis* (panel G) have a very high variance, likely due to poorer sample quality.

To assess the overall change in the oral bacteriome and virome composition, we further performed Principal Coordinates Analysis (PCoA). As shown in Fig. 8A, we found that PC3 and PC4 separate the control group from the infection group (explained variance of 9.6% and 8.3%, respectively. See also Supplementary Fig. 1 for the first 5 PCs). To investigate if the microbiome compositions of other groups were more similar to the control group or the infection group, we further overlaid each of the other groups on top of the control group and the infection group. Importantly, we found that among infected animals, those treated with nisin (Fig. 8B) and the nisin-producing *L. lactis* probiotic (Fig. 8C) were similar to the control group, indicating that nisin and *L. lactis* drive the microbiome composition toward the healthy state. In contrast, those treated with the non-nisin producing *L. lactis* (Fig. 8D) were in between the control and infection group, indicating that non-nisin producing *L. lactis* is less effective as a treatment in shifting the oral microbiome toward the healthy control. Other non-infection groups were more similar to the control group (nisin in Fig. 8E, *L. lactis* in Fig. 8F), except the non-nisin producing *L. lactis* group (Fig. 8G), which had a high variance.

**Fig. 9:**
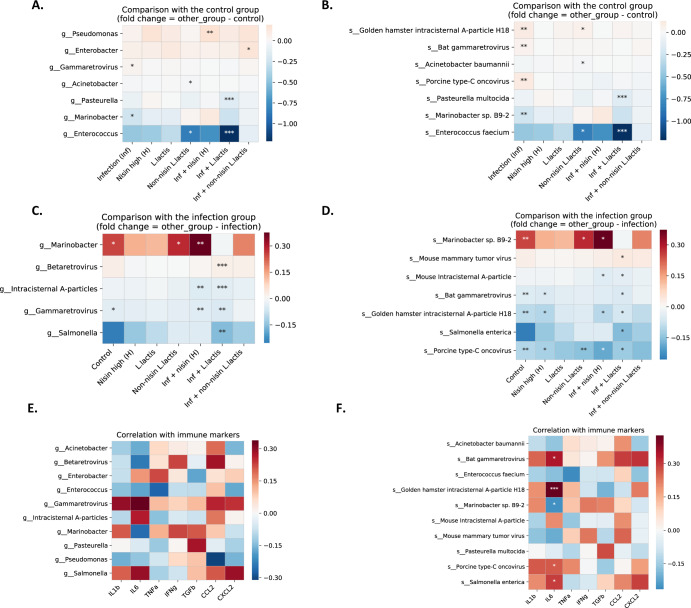
Differential abundance analysis for bacteria and viruses across groups highlight bacterial and viral members that align with healthy controls or infection and correlate with cytokine levels. The groups included Control, Infection, Nisin (H), *L. Lactis*, Non-nisin *L. lactis*, Infection + nisin (H), Infection + *L. lactis*, and Infection + Non-nisin *L. lacti*s. **A** Comparison at genus level between the Control group and other groups. **B** Comparison at species level between the Control group and other groups. **C** Comparison at genus level between the Infection group and other groups. **D** Comparison at species level between the Infection group and other groups. The color gradient represents the fold-change against the reference group (**A** and **B**; Control group, and **C** and **D**; Infection group). Red color means positive fold-change, blue color means negative fold-change and white color means no change. The Pearson’s correlation was computed with a *p*-value based on *t*-test. Asterisks represent the significant level; ***FDR < 0.1, **FDR < 0.2, FDR* < 0.3. **E** and **F** A correlation of all significant microbes (genus and species significant in at least one comparison in Fig. 9) with immune cytokine levels were computed across all animals. **E** Correlation of significant genus members with immune cytokine levels. **F** Correlation of significant species members with immune cytokine levels. The Benjamini–Hochberg procedure was performed for multiple testing for each immune marker (across all microbial species) separately. The color gradient represents the type of correlation (red means positive, blue means negative and white means no correlation) and the asterisks represent the level of significance; ***FDR < 0.1, **FDR < 0.2, FDR* < 0.3.

Furthermore, we identified bacteria and viruses at the genus and species level that showed differences in abundance across groups. In this regard, in order to identify specific differences, we performed two different analyses; the first analysis was based on using the healthy control group as the reference group (Fig. 9A, B) and a second analysis was based on using the infection group as the reference group (Fig. 9C, D).

At the genus level and looking at differences relative to the control group, we observed that the genus *Enterococcus* was in lower abundance across different groups when compared against the control group (Fig. 9A). This was specifically observed in the infection plus *L. lactis* (FDR < 0.1) and non-nisin *L. lactis* groups (FDR < 0.3) (Fig. 9A). The infection plus nisin group also showed a lower abundance, although this did not reach statistical significance. In addition, the genus *Marinobacter* showed a reduced abundance in the infection group (FDR < 0.3) compared to the control. Moreover, the genus *Pasteurella* showed a reduced abundance in the infection plus *L. lactis* group (FDR < 0.1) compared to the control. Also, the genus *Pseudomonas* and genus *Enterobacter* showed an increased abundance compared to the control, specifically in the infection plus nisin (FDR < 0.2) and infection plus non-nisin *L. lactis* (FDR < 0.3) groups, respectively (Fig. 9A). The groups with the least change relative to the control group were the nisin and *L. lactis* groups.

At the species level and looking at differences relative to the control group (Fig. 9B), we observed that the *Golden Hamster Intracisternal A-particle H18*, *Bat gammaretrovirus* and *Porcine type-C oncovirus* showed increased abundance (FDR < 0.2) upon infection compared to the control group, suggesting their role in the disease process. However, *Marinobacter sp. B9-2* showed a reduced abundance (FDR < 0.3) in the infection group compared to the control group, suggesting its role in health. In addition, both *Enterococcus faecium* and *Pasteurella multocida* showed a decreased abundance (FDR < 0.1) in the infection plus *L. lactis* group compared to the control group. The non-nisin producing *L. lactis* group showed a decreased abundance of *Enterococcus faecium* and an increased abundance of *Golden Hamster Intracisternal A-particle H18* (FDR < 0.3) compared to the control group.

At the genus level and looking at differences relative to the infection group (Fig. 9C), we observed that the infection plus *L. lactis* group showed decreased abundances at the genus level for *Salmonella* (FDR < 0.2), *Gammaretrovirus* (FDR < 0.2), *Intracisternal A-particles* (FDR < 0.1) compared to the infection group. However, *Betaretrovirus* (FDR < 0.1) showed an increased abundance in the infection plus *L. lactis* group compared to the infection group. The infection plus nisin group also showed a decreased abundance in *Gammaretrovirus* (FDR < 0.2) and *Intracisternal A-particles* (FDR < 0.2) compared to the infection group. In addition, the genus *Marinobacter* showed a higher abundance across different treatment groups compared to the infection group, again suggesting a potential association with health.

At the species level and looking at differences relative to the infection group (Fig. 9D), we also observed some microbes in higher abundance across different groups compared to the infection group (e.g., *Marinobacter sp. B9-2* and *Mouse mammary tumor virus*); suggesting their potential involvement in maintaining health. In contrast, *Mouse intracisternal A-particle*, *Bat-gammaretrovirus, Golden hamster intracisternal A-particle H18, Salmonella enterica*, and *Porcine type-C oncovirus* were in lower abundance across different groups compared to the infection group, suggesting their potential involvement in the transition to disease (Fig. 9D).

### Specific microbial species (bacteria and viruses) are correlated with IL-6 levels

We next examined potential correlations between microbial changes and cytokine levels across all groups. We identified significant correlations between specific microbial species (bacteria and viruses) and IL-6 levels (Fig. 9E, F). Specifically, *s_Golden hamster intracisternal A-particle H18* (FDR < 0.1) exhibited the highest level of positive correlation with IL-6 levels. The following species also exhibited a positive correlation with IL-6 but at a decreased level of significance (FDR < 0.3): *s_Bat gammaretrovirus, s_Salmonella enterica*, and *s_Porcine type-C oncovirus*. The following, *s_Marinobacter sp.B9-2*, was the only species showing a moderate negative correlation (FDR < 0.3) with IL-6 levels.

## Discussion

Studies exploring the potential of probiotics to suppress periodontal pathogens or anaerobic bacteria in human and animal studies have shown some benefits. Human studies exploring probiotics as monotherapy or adjunctive therapy have shown some benefit or neutral effects in reducing periodontal pathogens or anaerobes with the probiotics *Lactobacillus salivarius WB21*^49^, *L. reuteri*^20,27,50,51^, bacillus^52^, *L. plantarum*^35^*, L. rhamnosus* SP1^53^, *B. lactis*^33^, and various *Streptococci*^26,34,54^. Animal studies also showed some benefits. When *Lactobacillus brevis* or *Bifidobacterium lactis* were applied in a murine model of periodontitis, there was a significant decrease in the counts of anaerobic bacteria relative to aerobic bacteria^55,56^. Use of *Lactobacillus rhamnosus GG* showed no antimicrobial activity against *P. gingivalis* and *F. nucleatum*^57^. The current investigation demonstrated that the nisin-producing probiotic and nisin itself reduced the oral levels of three important periodontal pathogens: *P. gingivalis, T. forsythia*, and *F. nucleatum*, indicating nisin’s/nisin-producing probiotic’s efficacy in consistently removing these pathogens from oral surfaces. In addition, the results showed that the four periodontal pathogens (*P. gingivalis, T. forsythia, F. nucleatum, T. denticola)* were detected in oral swabs of uninfected mice at baseline; potentially because of false-positive results due to the highly sensitive qRT-PCR methods employed or contamination. Some recent studies suggest that it is also possible that these periodontal pathogens harbor in this kind of murine species, which has not been found before. Wu et al.^47^ reported that the genus Staphylococcus, Klebsiella, and Rothia were found in healthy oral microbiomes of rats by 16S rDNA high-throughput sequencing, and some Staphylococcus spp., Klebsiella spp., and Rothia spp. pathogens and opportunistic pathogens were present in the oral microbiome of rats. *Prevotella intermedia*, which is a periodontal pathogen in the human cavity, was detected in healthy canine oral samples by next-generation sequencing^48^. These studies suggest the possible existence of pathogens in healthy oral microbiomes of animals. More advanced sequencing techniques, such as metagenomics, may be useful in confirming these results.

The independent effects of stand-alone probiotic therapy on periodontal bone loss have not been examined in humans, however, studies in animal models (dogs, rats, mice) have shown significant beneficial effects with use of various *Streptococci* species*, Bacillus subtilis, Lactobacillus brevis, Saccharomyces cerevisiae, Bacillus subtilis, Bacillus licheniformis, Bifidobacterium animalis subsp. lactis, Bifidobacterium lactis, and Lactobacillus rhamnosus GG*^26,55–63^. In the current study, significant decreases in alveolar bone loss and intrabony defect formation were observed with the use of the nisin-producing *Lactococcus lactis* probiotic or nisin itself. Furthermore, low and high concentrations of nisin were equally effective at reducing bone loss.

Probiotics have not been examined for their potential to reduce the host systemic antibody response to periodontal pathogens in a periodontal setting in humans or animals^26^. The current investigation revealed that the *Lactococcus lactis* probiotic or nisin itself can significantly reduce the systemic antibody response to all periodontal pathogens. This suggests that this nisin-producing probiotic and nisin have significant potential for blocking the negative downstream systemic effects associated with these periodontal pathogens. It is noteworthy that the non-nisin producing *L. lactis* also mediated some beneficial effects. Some of the partial effects mediated by the non-nisin producing *L. lactis* control may be due to it is inherent properties as a lactic acid bacteria (low pH and enzymatic activity); which may contribute to its effects^36,64^. For example, the non-nisin producing probiotic was also able to decrease the antibody response to the periodontal pathogens, however the effect was not as significant as the nisin-producing *L. lactis* (Fig. 4).

The host immune and extracellular matrix/bone turnover response to probiotics have been examined in humans and animal studies. In humans, *Lactobacillus casei, Lactobacillus reuteri*, and *Lactobacillus brevis*, reduced the levels of MMP-3, elastase, and cytokines and chemokines, including TNF-α, IL-8, IL-1β, PGE_2_^26,65–68^ when used as monotherapy. When used as adjunctive therapy, the probiotics *Lactobacillus reuteri* and *Bifidobacterium lactis* reduced the levels of pro-inflammatory cytokines, including TNF-α, IL-1β, IL-17, IL-8, increased the levels of anti-inflammatory cytokines, including IL-10, and improved the levels of ECM molecules (MMP-8 and TIMP-1)^28,33,69^. In animals (rodents), *Lactobacillus brevis, Saccharomyces cerevisiae, Bacillus subtilis, Bacillus licheniformis, Lactobacillus casei subspecies pseudoplantarum, Lactobacillus casei subsp casei, Lactobacillus fermentum, Lactobacillus helveticus*, and *Bifidobacterium animalis subspecies lactis* reduced the levels of pro-inflammatory cytokines, including TNF-α, IL-1β, IL-6, and IL-17A, increased the levels of anti-inflammatory cytokines, decreased the level of inflammatory cells and bone turnover markers, including C-terminal telopeptide, TRAP signal/TRAP-positive osteoclasts, nuclear factor-κB ligand (RANKL)/osteoprotegerin (OPG) ratio^55–57,60–63,70^. Similarly, in the current study, the probiotic *L. lactis* and its nisin bacteriocin decreased both the number of oral inflammatory cells and the number of pro-inflammatory cytokines.

The novel discovery that nisin and the nisin-producing probiotic *L. lactis* promoted increases in the number of tissue reparative cells of the periodontium is surprising. Pazzini et al found that probiotic therapy promoted a decrease in the number of osteoclasts in the tissues around teeth submitted to mechanical loading^71^. Parvaneh also reported that probiotic supplementation increased osteoblasts and decreased osteoclasts in a model of ovariectomized rats^72^. These studies suggested a role for probiotics in meditating cell proliferation, and a potential role in tissue and bone remodeling, but the potential for a probiotic or bacteriocin to promote the proliferation of host reparative cells in a periodontitis model has not been previously documented. In this study, we found that in response to the polymicrobial infection, nisin and/or the nisin-producing *L. lactis* probiotic enhanced the number of gingival fibroblasts, periodontal ligament cells, and bone lining cells; cells which are responsible for the wound healing and regenerative function of the tissue. Further research demonstrated that Ki-67 expression increased in human periodontal ligament cells upon nisin treatment in vitro. Ki-67 is a well-recognized nuclear proliferation marker, which is expressed in proliferating cells, during all active phases of the cell cycle. These findings have implications for the clinical sequelae of periodontal disease. Namely, in addition, to the aforementioned beneficial effects of nisin in mitigating periodontal disease bone loss and the host inflammatory response, while resetting the oral microbiome towards control levels, this additional finding highlights that nisin and a nisin-producing probiotic may promote a proliferative and reparative phenotype and tissue restitution following disease.

Limited studies have examined a probiotic’s ability to shift a disease-associated oral microbiome. In humans, one study found that lozenges containing *L. rhamnosus* GG and *Bifidobacterium animalis* mediated no change in the microbial composition of saliva using a focused oral microbe microarray^73^. One study in rats, using *Bifidobacterium animalis subspecies lactis* showed an increase in the levels of Actinomyces and *Streptococci*-like species while decreasing the levels of *Veillonella parvula*, *Capnocytophaga sputigena, Eikenella corrodens*, and *Prevotella intermedia*-like species^59^. Importantly, the current study revealed that the probioltic *L. lactis* and its bacteriocin nisin can shift a disease-associated oral bacteriome and virome back towards a healthier state (Fig. 5B, C). This agrees with our recent in vitro findings in oral biofilms that nisin and a nisin-producing probiotic shift periodontal pathogen-spiked oral biofilms back towards a control/healthy state^44^. Maintaining or promoting a healthy microbiome in the course of treatment with probiotics is being recognized as an important parameter that should be evaluated^74–76^. In this study, we used the approach of identifying the complex microbial signature of periodontal health as a baseline for comparison to evaluate and confirm a restitution of “health” following antimicrobial treatment for periodontal disease.

A long-standing premise in the pathogenesis of periodontal disease has been its association with pathogenic bacteria, especially members of the so-called Red Complex. The current study and others highlight the importance of new and emerging microbes, both bacteria and viruses, in periodontal disease pathogenesis^12,77–82^ and their potential shift with treatment^78,83^. These microbes may be important signatures useful in monitoring treatment and to determine shifts that signify health. We observed that the species *Marinobacter sp. B9-2* was in higher abundance in the healthy control group compared to the infection group. However, three viruses, *Golden Hamster Intracisternal A-particle H18, Bat gammaretrovirus*, and *Porcine type-C oncovirus* showed an increased abundance (FDR < 0.2) in the infection group compared to the control group and also relative to other treatment groups (Fig. 6B). Thus, periodontal health was associated with *Marinobacter sp. B9-2*, whereas the three viruses, *Golden Hamster Intracisternal A-particle H18, Bat gammaretrovirus*, and *Porcine type-C oncovirus*, were associated with periodontal disease. These findings are consistent with our earlier observations showing that these three viral infection-associated microbes were also associated with bone loss, whereas *Marinobacter* decreased with bone loss^12^. Treatment generally shifted microbes towards the healthy control. The significance of these specific microbes and their role in health and disease and response to treatment has not been previously described. *Marinobacter* is a genus of *Proteobacteria* found in sea water and a number of strains and species can degrade hydrocarbons^84^. *Intracisternal type A particles* are defective retroviruses in rodent genomes^85^. *Bat gammaretrovirus* are retroviruses that can cause malignancies and immune deficiencies in mammals, reptiles and birds^86^. *Porcine type-C oncovirus* is a type of *gammaretrovirus* that lives in extreme environments and can be found in the human microbiome^87^. Further study is warranted to determine the relevance of these microbes in human oral health and disease.

Several of these microbial species were also significantly correlated with the cytokine host immune response. Namely, *s_Golden hamster intracisternal A-particle H18* (highest correlation), *s_Bat gammaretrovirus, s_Salmonella enterica*, and *s_Porcine type-C oncovirus* exhibited a significant correlation with IL-6 levels. However, *s_Marinobacter sp.B9-2* was significantly negatively correlated with IL-6 levels. These findings further highlight the tight relationship between the microbiome and the host immune response; an interaction well known in conditions of health and disease^88^.

## Conclusions

In summary, this study highlights an approach to realign the oral microbial dysbiosis of periodontal disease and its related sequalae (bone loss, altered host immune response) towards health. Treatment with antibiotics and probiotics have been used to modulate the microbial, immunological, and clinical landscape of periodontal disease with some success. Antibacterial peptides or bacteriocins, such as nisin, and nisin-producing probiotics, such as *Lactococcus lactis*, have not been examined in this context. However, they warrant examination because of their well characterized biomedical benefits in eradicating biofilms and oral pathogenic bacteria, while also modulating immune mechanisms. This study demonstrates that nisin and nisin-producing probiotic treatment inhibit periodontal disease-related bone loss and host immune responses while significantly shifting the oral bacteriome and virome towards the healthy control state. This shift was characterized by a unique signature where health was associated with a *Proteobacteria* (*Marinobacter sp. B9-2*), whereas three retroviruses (*Golden Hamster Intracisternal A-particle H18, Bat gammaretrovirus*, and *Porcine type-C oncovirus*) were associated with disease. The ability to shift the oral microbiome towards health may be a useful approach to treating periodontal disease in vivo. Further, the novel discovery that nisin and a nisin-producing probiotic promote the numbers of host reparative cells reveals a potentially new biomedical application for nisin in tissue and bone remodeling. Nisin’s ability to shift dysbiotic microbiomes towards health, mitigate the tissue breakdown and host response associated with chronic polymicrobial diseases, and promote a proliferative phenotype, may benefit chronic inflammatory diseases, like periodontal disease and negate the systemic effects associated with the disease.

## Methods

### Periodontal bacteria and polymicrobial inoculum

The following periodontal pathogens were tested, *P. gingivalis* FDC 381, *T. denticola* ATCC 35405, *T. forsythia* ATCC 43037, and *F. nucleatum* ATCC 10953. They were cultured as described previously^12,87^.

For the oral polymicrobial infection, the four periodontal pathogens were prepared and mixed as previously described and used for the oral inoculation^12,89^.

### *Lactococcus lactis* growth conditions

Two *L. lactis* strains were used in this study; nisin-producing *L. lactis* (ATCC 11454) was obtained from ATCC and non-nisin producing *L. lactis* (NZ9800) was kindly provided by Dr. Paul Cotter, Head of the Food Biosciences Department in the Teagasc Food Research Center, Cork Institute of Technology, Ireland. *L. lactis* ATCC 11454 produces nisin A as reported before^90^. *L. lactis* strains were grown in Brain Heart Infusion (BHI, Sigma-Aldrich) media overnight in a 37 °C shaking incubator. The *L. lactis* strains were then pelleted by centrifugation, resuspended in phosphate-buffered saline (PBS) to a concentration of 1 × 10^10^ CFU/ml, and mixed with an equal volume of sterile 4% CMC. This mixture was used for oral inoculation.

### Nisin preparation

An ultra-pure (>95%) food grade form of nisin Z (NisinZ^®^ P) also referred to as nisin ZP was purchased from Handary (S.A., Brussels, Belgium), a primary manufacturer of nisin in the food industry. From here forward, nisin ZP will be referred to as nisin. The stock solution was prepared at a concentration of 600 or 200 μg/ml in sterile water, filter sterilized, and stored at 4 °C for a maximum of 5 days for use in experiments. For oral treatment of mice, the nisin solution was mixed with an equal volume of sterile 4% CMC to reach the final concentration (300 or 100 μg/ml).

### Infection and treatment of mice

A total of 60 8-week-old BALB/cByJ female mice (The Jackson Laboratories, Bar Harbor, ME) were housed in microisolator plastic cages and randomly distributed into ten groups (six mice per group). Sample size was based on our previous publication, which measured similar outcome variables that revealed significant differences in all measured parameters^12^. The description of the experimental groups and infection and treatment protocols are shown in (Fig. 1A, B). The experimental protocols were approved by the Institutional Animal Care and Use Committee of the University of California, San Francisco (IACUC APPROVAL NUMBER: AN171564-01B). In an effort to reduce the number of animals per the requirements of the IACUC and to follow best practices for the use of animals in experimentation, we used the same Control and Infection groups as in our previous study. All the mice were given trimethoprim (0.17 mg per ml) and sulfamethoxazole (0.87 mg per ml) daily for 7 days in the drinking water and their oral cavity was rinsed with 0.12% chlorhexidine gluconate (Peridex) mouth rinse to inhibit the native oral microbiota^12,14^. The polymicrobial inoculum (5 × 10^9^ combined bacteria per ml; 1 × 10^9^ cells in 0.2 ml per mouse; 2.5 × 10^8 ^*P. gingivalis*, 2.5 × 10^8 ^*T. denticola*, 2.5 × 10^8 ^*T. forsythia* and 2.5 × 10^8 ^*F. nucleatum*) was administered topically in the morning for 4 consecutive days every week for a total of 8 weeks. Nisin (100 or 300 μg/ml, 0.2 ml per mouse) and *L. lactis* (5 × 10^9^ bacteria per ml; 1 × 10^9^ cells in 0.2 ml per mouse) were administered every day in the evening every week for a total of 8 weeks. A sterile 2% CMC solution was administered as the control treatment.

Following 8 weeks of polymicrobial infection, oral swab samples were collected with a sterile micro sized cotton swab to evaluate the microbial status and to examine the effect of nisin on periodontal pathogens. Teeth and surrounding gingival tissue were swabbed and the cotton tip was immersed in 10:1 Tris-EDTA buffer immediately and stored at –80 °C until further processing for DNA isolation. Then mice were euthanized and blood was collected for analysis of antibody response to the periodontal pathogens. Maxillae and mandibles were resected from each mouse for morphometric, histologic, immunologic, and sequencing analysis. Where possible measurements were performed in blinded fashion; for example two blinded examiners (experienced periodontists) performed all bone loss measurements twice at separate times.

### DNA isolation from oral swabs, ethanol precipitation, and real-time PCR to confirm bacterial infection

DNA isolated from oral swabs was used to evaluate and confirm infection with the periodontal pathogens in the mice using methods described in our previous study^12^.

### Morphometric analysis of periodontal alveolar bone loss

After autoclaving and de-fleshing to remove all the soft tissues, the left maxillae and mandible from each mouse were processed to evaluate alveolar bone loss and intrabony defects as previously described in our study^12,14^.

### Histopathological evaluation of periodontal inflammation and cellular content

The right maxilla was resected from each mouse and immediately fixed in 4% paraformaldehyde for 24 h, then decalcified with diethyl pyrocarbonate-treated 0.5 M ethylenediaminetetraacetic acid (pH 8) for 28 days at room temperature. The decalcified specimens were then dehydrated and embedded in paraffin using a fully-enclosed tissue processor (ASP300S, Leica Biosystems, Buffalo Grove, IL, USA). Tissue blocks were cut into serial sections (4 μm) parallel to the mesiodistal plane using a microtome, then sections were stained with Mayer’s hematoxylin (Sigma-Aldrich, St. Louis, MO, USA) and eosin Y solution (Sigma-Aldrich) for assessment of inflammation. The sections were examined with a stereomicroscope.

The number of inflammatory cells (round-shaped nuclei) and gingival fibroblast (spindle-shaped nuclei) within a square field (100 × 100 μm) in connective tissue adjacent to the gingival epithelium between first and second molars were morphologically evaluated and counted in three tissue sections per mouse specimen (*n* = 3 per group). Similarly, the number of periodontal ligament (PDL) cells (spindle-shaped nuclei in the PDL space) and alveolar bone lining cells (cell nuclei on bone surface) were counted. All cell counts were averaged for each group, and data were expressed as the mean number of cells per 1.0 mm^2^ of connective tissue in the maxillary specimens.

### Effects of nisin on human periodontal ligament cell proliferation and Ki-67 gene expression in vitro

Approval to conduct human subjects’ research, including protocols for the collection and use of human teeth and periodontal ligament (PDL) tissue was obtained from the University of California San Francisco Institutional Review Board (#16–20204; reference #227030). Consent was not obtained due to anonymity of the samples.

Human periodontal ligament (hPDL) cell primary culture was carried out as previously described^91,92^. For the proliferation assays, 0.8 × 10^4^ hPDL cells were seeded on 96-well plates. The next day, cells were treated with 0 (control), 10, 20, 30, 40, 50, 100, 150, or 200 µg/ml of nisin diluted in MEM-α (Gibco, USA) for 24 h. The subsequent day, hPDL cell proliferation rate was determined using the CyQUANT NF Cell Proliferation Assay according to the manufacturer’s instructions and the resulting fluorescence was measured using a Spectramax M2 microplate spectrophotometer (Molecular Devices, USA).

For the Ki-67 gene expression analysis, 2.4 × 10^5^ hPDL cells were seeded onto 6-well plates. The next day, cells were treated with 0 (control), 50 or 100 µg/ml of Nisin in MEM-α (Gibco, USA) for 24 h. The subsequent day, hPDL cells were washed with PBS and total RNA was extracted using the RNeasy mini kit (Qiagen, Germany) according to the manufacturer’s instructions, and the RNA yield was quantified using a NanoDrop UV–Vis Spectrophotometer (Thermo Scientific, USA). Next, RNA Reverse Transcription into cDNA was performed using the SuperScript III vilo cDNA synthesis kit (Invitrogen, USA). Samples were analyzed on a Bio-Rad MyCycler Thermal Cycler according to manufacturer’s instructions. cDNA samples were, then, probed for Ki-67 gene expression via qPCR using TaqMan Gene Expression Assays (Thermo Scientific, USA–Assay ID Hs00606991_m1) on a QuantStudio 3 platform (Applied Biosystems, USA). The relative expression levels of the target gene were plotted as fold-change compared with the untreated or negative controls. The 2^–ΔΔCT^ method was used to normalize the Ki-67 expression against GAPDH (Thermo Scientific, USA–Assay ID Hs02786624_g1) expression and determine relative changes in Ki-67 gene expression.

### PCR evaluation of immune cytokine profiles from gingival tissues

Mouse gingival tissue was treated overnight at 4 °C with RNA stabilization solution (RNAlater, Invitrogen) after tissue harvesting. Samples were powdered with a mortar and pestle under continuous liquid nitrogen, and total RNA was then isolated from each sample using the RNeasy mini Kit (QIAGEN). The purity and quantity of the RNA were evaluated using the NanoVue Plus spectrophotometer (Biochrom Ltd.). Subsequently, total RNA was synthesized into cDNA using the SuperScript VILO Master Mix (11755050; Invitrogen).

To assess the immune cytokine profiles in gingival tissues, relative gene expression was evaluated by real-time PCR as in our previous study^93^ using the following TaqMan primers and probes (TaqMan Gene Expression Assays; Applied Biosystems): interleukin-1β (IL-1β; Mm00434228_m1), IL-6 (Mm00446190_m1), tumor necrosis factor-α (TNF-α; Mm00443258_m1), interferon gamma (IFN-γ; Mm01168134_m1), C-C Motif Chemokine Ligand 2 (CCL2; Mm00441242_m1), C-X-C Motif Chemokine Ligand 2 (CXCL2; Mm00436450_m1), and transforming growth factor beta 1 (TGF-β1; Mm01178820_m1). Glyceraldehyde 3-phosphate dehydrogenase (GAPDH; Mm99999915_g1) was used as a housekeeping gene to normalize the amount of mRNA present in each reaction. PCR was performed in 20 μl reaction mixtures containing the TaqMan Fast Advanced Master Mix, cDNA template (20 ng/well), primers, and probes using a QuantStudio 3 Real-Time PCR system (Thermo Fisher Scientific). The optimized thermal cycling conditions were as follows: 20 min at 95 °C, followed by 40 cycles per 1 min at 95 °C, and 20 min at 60 °C. To compare the expression levels among different samples, the relative expression level of the genes was calculated by the comparative CT (ΔΔCT) method using QuantStudioTM Design & Analysis Software.

### Serum antibody analysis

Serum from all 60 mice was collected on the day of euthanasia and used to determine the host response in the form of immunoglobulins (IgG) against *P. gingivalis*, *T. denticola*, *T. forsythia*, and *F. nucleatum* by an enzyme-linked immunosorbent assay (ELISA) as previously described^12,14^.

### DNA isolation from gingival tissue for next-generation shotgun sequencing

DNA was extracted from the mandibular gingival tissue of all mice (6 mice/group) using the QIAamp® DNA Mini kit (Qiagen, Germantown, MD, USA) as follows. The gingival tissue was ground in liquid nitrogen with a mortar and pestle and 180 μl of Buffer ATL was mixed with 25 mg of tissue powder by vortexing. Then, 20 μl of QIAGEN proteinase K was applied to each sample and samples were incubated at 56 °C for 3 h in a shaking water bath. Subsequently, 20 μl of the RNase reagent (20 mg/ml) was added to the samples followed by incubation for 2 min at room temperature. After adding 200 μl of Buffer AL, the samples were incubated at 70 °C for 10 min. In addition, 200 μl of pure ethanol was mixed with each sample. This entire mixture was then applied into the QIAamp Mini spin column and centrifuged at 6000 × *g* for 1 min. Next, 500 μl of Buffer AW1 were added to the spin column and samples centrifuged at 6000 × *g* for 1 min. Then, 500 μl of Buffer AW2 was added and samples were centrifuged at full speed (20,000 × *g*) for 3 min, followed by centrifugation (20,000 × *g*) for 1 min again to eliminate the chance of possible Buffer AW2 carryover. Lastly, samples were incubated with 200 μl of Buffer AE in the spin column, which was placed in a clean 1.5 ml microcentrifuge tube at room temperature for 5 min, then DNA were eluted by centrifugation at 6000 × *g* for 1 min.

The purity and quantity of the DNA were evaluated using the NanoDrop™ OneC Microvolume UV–Vis Spectrophotometer (Thermo Scientific), which met quality control measures for subsequent shotgun sequencing analysis.

### Metagenome shotgun sequencing and microbiome data production and analyses

Shotgun metagenomic sequencing library preparation was performed by Novogen, Inc. The libraries were prepared according to a standard protocol from Illumina, and at least 1 Gb of 150 bp pair-end reads per sample were sequenced on the Illumina Hiseq4000 machines. FASTQ files were generated from the sequencing machines and used for the analyses of the bacteriome/microbiome and virome as described below.

### Data processing

The following criteria were used for processing and cleaning up the raw data. Low quality bases (*Q*-value ≤ 38), which exceeded a certain threshold (40 bp by default) were trimmed. Reads which contained N nucleotides over a certain threshold (10 bp by default) were trimmed. Reads which overlapped with adapter over a certain threshold (15 bp by default) were trimmed.

### Metagenome assembly

We utilized de novo assembly for each sample as follows. Samples passing quality control were assembled initially using SOAPdenovo (http://soap.genomics.org.cn/soapdenovo.html). The Scaffolds were cut off at “N” to get fragments without “N”, called Scaftigs. Clean data for all samples were then mapped to assembled Scaftigs using SoapAligner (http://soap.genomics.org.cn/soapaligner.html) and unutilized paired-end reads were collected. Mixed assembly was conducted on the unutilized reads with the same assembly parameter. The scaftigs of each sample and mixed assembly, which were less than 500 bp, were trimmed.

### Taxonomy annotation

The following taxonomy annotation scheme was used. We aligned unigenes to the NCBI nonredundant database with DIAMOND to taxonomically annotate each metagenomic homolog (MEGAN). According to the abundance table of each taxonomic level, various analyses were performed using custom scripts by R and Python.

### Statistical analysis

SPSS 21.0 statistical software (IBM, Chicago, IL, USA) was used for statistical analysis of the non-sequencing data. Student’s *t*-test was used to compare two independent groups. For comparison of intrabony defects, data were expressed as frequency and percentage, and a chi-square test was used for analysis. Further, analyses of the PCR data from the oral swabs and quantification of inflammatory cells were performed using an ANOVA followed by a Tukey’s test. Data were presented as means ± standard deviations (SD). Values of *p* < 0.05 were considered significant.

For the microbiome/virome analyses, we normalized the data to have 1 million reads per sample (reads per million, RPM). We filtered out taxa with average read counts less than 1 RPM per standard protocols. We removed five samples, namely Infection 1, Infection 6, Non-nisin *L. lactis* + Infection 4, Non-nisin *L. lactis* + Infection 6 and Nisin + Infection 3, that have low sequencing coverage. We used the Shannon diversity index to quantify bacterial and viral diversity across different groups. In order to compare the difference of bacterial contents, viral contents, and Shannon diversities between different groups, we computed the *p*-values using a two-sample *t*-test assuming equal variance of samples from the two groups. For the Principal Coordinates Analysis (PCoA), we further restricted to species with RPM < 500 to avoid the result being dominated by commonly present species. Three species, namely Mouse Intracisternal A-particle, Chlamydia abortus, Chlamydia trachomatis, were filtered out under this criterion. We used the Bray Curtis dissimilarity to quantify the difference between microbiome composition of different samples. The 95% confidence ellipses were computed assuming that the data in each group followed a two-dimensional normal distribution. For the differential abundance analysis, we performed a log transformation (log10 (RPM + 0.1)) for the bacterial and viral read counts and used a two-sample *t*-test to compute the *p*-values, assuming equal variance in the two groups. We further used the Benjamini–Hochberg procedure^94^ to correct for multiple comparisons. We reported the corresponding false discovery rate (FDR) for conducting pair-wise comparisons (e.g., Infection versus Control), and the multiplicity is the total number of taxa. For correlating microbial species with the immune markers, we considered the data in log space for both read counts and immune marker measurements (log10(*x* + 0.1)). We considered only microbial species that are significant in at least one differential abundance comparison (comparison vs. control or vs. infection). We computed Pearson’s correlation with a *p*-value based on *t*-test. We performed the Benjamini–Hochberg procedure^94^ for multiple testing for each immune marker (across all microbial species) separately.

### Reporting summary

Further information on research design is available in the Nature Research Reporting Summary linked to this article.

## Supplementary information

Supplemental Figure

Reporting Summary Checklist

## Data Availability

The data and code are available at https://figshare.com/articles/dataset/Nisin_Probiotic_Prevents_Periodontal_Disease_and_Inflammation_while_Promoting_Periodontal_Regeneration_and_Shift_Toward_Healthy_Microbiome_Virome/14237963
