# Supplementary material for: Nisin probiotic prevents inflammatory bone loss while promoting reparative proliferation and a healthy microbiome
Source: NPJ Biofilms Microbiomes. 2022 Jun 7;8:45. doi: 10.1038/s41522-022-00307-x (PMC9174264; doi:10.1038/s41522-022-00307-x)
Supplement: Supplementary file 1 — Supplemental Figure [file 41522_2022_307_MOESM1_ESM.pdf]

Supplemental Figure 1

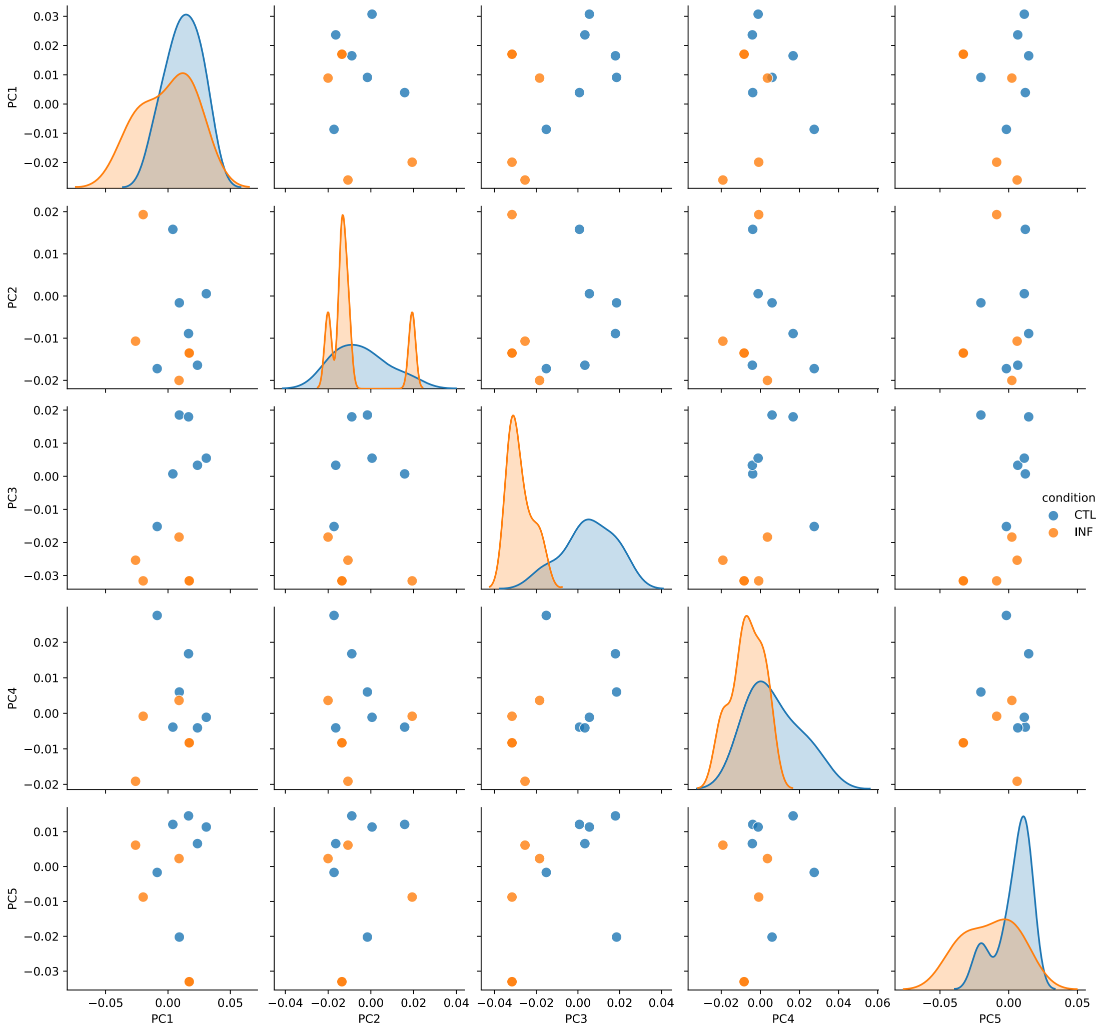

Supplemental Figure 1. PCoA plot of the first 5 PCs for the control and the infection group. PCoA plot indicate the variance of the oral bacteriome and virome composition across the control and infection groups. PC3 and PC4 separate the control group from the infection group (explained variance of 9.6% and 8.3%, respectively), and used for the further analysis. Each dot in the graph represents individual data points from each mouse in the control and infection groups.
