# Supplementary material for: Nisin probiotic prevents inflammatory bone loss while promoting reparative proliferation and a healthy microbiome
Source: NPJ Biofilms Microbiomes. 2022 Jun 7;8:45. doi: 10.1038/s41522-022-00307-x (PMC9174264; doi:10.1038/s41522-022-00307-x)
Supplement: Supplementary file 2 — Reporting Summary Checklist [file 41522_2022_307_MOESM2_ESM.pdf]

Corresponding author(s): NPJBIOFILMS-01573R

Last updated by author(s): Mar 5, 2022

Reporting Summary

Nature Portfolio wishes to improve the reproducibility of the work that we publish. This form provides structure for consistency and transparency in reporting. For further information on Nature Portfolio policies, see our Editorial Policies and the Editorial Policy Checklist.

Statistics

For all statistical analyses, confirm that the following items are present in the figure legend, table legend, main text, or Methods section.

n/a	Confirmed	
☐	☒	The exact sample size (n) for each experimental group/condition, given as a discrete number and unit of measurement
☐	☒	A statement on whether measurements were taken from distinct samples or whether the same sample was measured repeatedly
☐	☒	The statistical test(s) used AND whether they are one- or two-sided<br>*Only common tests should be described solely by name; describe more complex techniques in the Methods section.*
☐	☒	A description of all covariates tested
☐	☒	A description of any assumptions or corrections, such as tests of normality and adjustment for multiple comparisons
☐	☒	A full description of the statistical parameters including central tendency (e.g. means) or other basic estimates (e.g. regression coefficient) AND variation (e.g. standard deviation) or associated estimates of uncertainty (e.g. confidence intervals)
☒	☐	For null hypothesis testing, the test statistic (e.g. F, t, r) with confidence intervals, effect sizes, degrees of freedom and P value noted<br>*Give P values as exact values whenever suitable.*
☒	☐	For Bayesian analysis, information on the choice of priors and Markov chain Monte Carlo settings
☐	☒	For hierarchical and complex designs, identification of the appropriate level for tests and full reporting of outcomes
☐	☒	Estimates of effect sizes (e.g. Cohen's d, Pearson's r), indicating how they were calculated

Our web collection on statistics for biologists contains articles on many of the points above.

Software and code

Policy information about availability of computer code

Data collection	Shotgun metagenomic sequencing library preparation was performed by Novogen, Inc. The libraries were prepared according to a standard protocol from Illumina, and at least 1 Gb of 150 bp pair-end reads per sample were sequenced on the Illumina Hiseq4000 machines. FASTQ files were generated from the sequencing machines and used for the analyses of the bacteriome/microbiome and virome.
Data analysis	SPSS 21.0 statistical software (IBM, Chicago, IL, USA) was used for statistical analysis of the non-sequencing data.<br>For sequencing data: Samples passing quality control were assembled initially using SOAPdenovo (http://soap.genomics.org.cn/soapdenovo.html). The Scaffolds were cut off at "N" to get fragments without "N", called Scaftigs. Clean data for all samples were then mapped to assembled Scaftigs using SoapAligner (http://soap.genomics.org.cn/soapaligner.html) and unutilized paired-end reads were collected.<br>We aligned unigenes to the NCBI nonredundant database with DIAMOND to taxonomically annotate each metagenomic homolog (MEGAN).

For manuscripts utilizing custom algorithms or software that are central to the research but not yet described in published literature, software must be made available to editors and reviewers. We strongly encourage code deposition in a community repository (e.g. GitHub). See the Nature Portfolio guidelines for submitting code & software for further information.

Data

Policy information about availability of data

All manuscripts must include a data availability statement. This statement should provide the following information, where applicable:
- Accession codes, unique identifiers, or web links for publicly available datasets
- A description of any restrictions on data availability
- For clinical datasets or third party data, please ensure that the statement adheres to our policy

The data and code are available at
https://figshare.com/articles/dataset/
Nisin_Probiotic_Prevents_Periodontal_Disease_and_Inflammation_while_Promoting_Periodontal_Regeneration_and_Shift_Toward_Healthy_Microbiome_Virome/
14237963

Field-specific reporting

Please select the one below that is the best fit for your research. If you are not sure, read the appropriate sections before making your selection.

☒ Life sciences ☐ Behavioural & social sciences ☐ Ecological, evolutionary & environmental sciences

For a reference copy of the document with all sections, see nature.com/documents/nr-reporting-summary-flat.pdf

Life sciences study design

All studies must disclose on these points even when the disclosure is negative.

Sample size	Sample size was based on our previous publication, which measured similar outcome variables that revealed significant differences in all measured parameters
Data exclusions	We filtered out taxa with average read counts less than 1 RPM per standard protocols. We removed 5 samples, namely Infection 1, Infection 6, Non-nisin L. lactis + Infection 4, Non-nisin L. lactis + Infection 6 and Nisin + Infection 3, that had low sequencing coverage.
Replication	All attempts at replication were successful; samples size replications were never less than 3.
Randomization	A total of 60 eight-week old BALB/cByJ female mice (The Jackson Laboratories, Bar Harbor, ME) were housed in microisolator plastic cages and randomly distributed into 10 groups (6 mice per group).
Blinding	Where possible measurements were performed in blinded fashion; for example two blinded examiners (experienced periodontists) performed all bone loss measurements twice at separate times.

Reporting for specific materials, systems and methods

We require information from authors about some types of materials, experimental systems and methods used in many studies. Here, indicate whether each material, system or method listed is relevant to your study. If you are not sure if a list item applies to your research, read the appropriate section before selecting a response.

Materials & experimental systems

n/a	Involved in the study
☐	☒ Antibodies
☒	☐ Eukaryotic cell lines
☒	☐ Palaeontology and archaeology
☐	☒ Animals and other organisms
☒	☐ Human research participants
☒	☐ Clinical data
☒	☐ Dual use research of concern

Methods

n/a	Involved in the study
☒	☐ ChIP-seq
☒	☐ Flow cytometry
☒	☐ MRI-based neuroimaging

Antibodies

Antibodies used	a mouse IgG ELISA quantification kit (Bethyl Laboratories, USA) was used; containing an alkaline phosphatase-conjugated goat anti-mouse IgG
Validation	A pilot test was first performed with serum diluted at 1:100 to confirm reactivity with the bacterial antigens.

Animals and other organisms

Policy information about studies involving animals; ARRIVE guidelines recommended for reporting animal research

Laboratory animals	eight-week old BALB/cByJ female mice (The Jackson Laboratories, Bar Harbor, ME) were used in the study
Wild animals	Not applicable
Field-collected samples	Not applicable
Ethics oversight	The experimental protocols were approved by the Institutional Animal Care and Use Committee of the University of California, San Francisco (IACUC APPROVAL NUMBER: AN171564-01B).

Note that full information on the approval of the study protocol must also be provided in the manuscript.

Yvonne Kapila
3/7/22
